# Bloodstream infections due to carbapenemase-producing Enterobacteriaceae in Italy: results from nationwide surveillance, 2014 to 2017

**DOI:** 10.2807/1560-7917.ES.2019.24.5.1800159

**Published:** 2019-01-31

**Authors:** Simone Iacchini, Michela Sabbatucci, Carlo Gagliotti, Gian Maria Rossolini, Maria Luisa Moro, Stefania Iannazzo, Fortunato D’Ancona, Patrizio Pezzotti, Annalisa Pantosti

**Affiliations:** 1 Department of Infectious Diseases, Istituto Superiore di Sanità, Rome, Italy; 2 European Programme for Public Health Microbiology Training (EUPHEM), European Centre for Disease Prevention and Control (ECDC), Stockholm, Sweden; 3 Agenzia Sanitaria e Sociale Regionale – Regione Emilia-Romagna, Bologna, Italy; 4 Careggi University Hospital, Florence, Italy; 5 Department of Experimental and Clinical Medicine, University of Florence, Florence, Italy; 6 Ufficio V – Prevention of Communicable Diseases and International Prophylaxis, Ministry of Health, Rome, Italy

**Keywords:** carbapenem-resistant Enterobacteriaceae, carbapenemase-producing Enterobacteriaceae, bloodstream infections, surveillance, Italy

## Abstract

Following the rapid increase of infections due to carbapenemase-producing Enterobacteriaceae (CPE) in Italy, the national surveillance of bloodstream infections (BSI) due to CPE (*Klebsiella pneumoniae* and *Escherichia coli*) was instituted in 2013. All CPE-BSI cases reported to the surveillance in the years 2014–17 were analysed in order to investigate incidence rate (IR), trend, main individual characteristics and enzymes involved in CPE resistance. Throughout this period, 7,632 CPE-BSI cases (IR: 3.14/100,000 inhabitants) were reported from all 21 regions and autonomous provinces in Italy, with an increasing number of reported cases (2014: 1,403; 2015: 1,838; 2016: 2,183; 2017: 2,208). CPE-BSI cases mainly occurred in subjects aged over 60 years (70.9%) and more frequently in males (62.7%) than in females. Most of the cases originated in hospitals (87.2%), mainly in intensive care units (38.0%), and were associated with central or peripheral venous catheter use (23.9%) or with urinary tract infections (21.1%). Almost all CPE-BSI (98.1%) were due to *K. pneumoniae* carrying the *K. pneumoniae* carbapenemase (KPC) enzyme (95.2%). These data show that carbapenemase-producing *K. pneumoniae* are endemic in our country, causing a high number of BSI and representing a threat to patient safety.

## Introduction

The increase in carbapenem resistance in Enterobacteriaceae through carbapenemase enzyme production has been reported worldwide [1-6]. Carbapenemase-producing Enterobacteriaceae (CPE) infections are difficult to treat since CPE are resistant to virtually all beta-lactam antibiotics and often contain additional mechanisms of resistance against second-line antibiotics such as aminoglycoside and fluoroquinolones. Recent studies have also shown emerging resistance to antibiotics of last resort (i.e. tigecycline or colistin), leaving very few therapeutic options [7-9]. According to the new taxonomy [10], carbapenem resistance is present not only in the family Enterobacteriaceae sensu stricto but also in the other families now belonging in the Enterobacterales order. When compared with carbapenem-susceptible infections, those due to carbapenem-resistant Enterobacterales (CRE) show higher mortality rates, especially for bloodstream infections (BSI) [11,12]. Resistance to carbapenems impacts considerably on healthcare costs and loss of productivity [13]. In England, the overall cost of an outbreak due to CPE involving 40 patients in five hospitals was estimated at ca EUR 1.1 million over a 10-month period between March and December 2015 [14]. The high occurrence of cases, dearth of alternative drugs, high mortality and economic burden make CPE infections an important threat to public health worldwide.

Data obtained from the European Survey on Carbapenemase-Producing Enterobacteriaceae (EuSCAPE) project showed low frequencies of CRE in many European countries. On average, 1.3 patients per 10,000 hospital admissions have carbapenemase-producing *Klebsiella pneumoniae* or *Eschericia coli* isolated from a clinical specimen [15]. Italy experienced a rapid increase of infections due to CRE and to CPE, mostly caused by *K. pneumoniae*. Data from the Italian surveillance of antibiotic resistance at the Istituto Superiore di Sanità (AR-ISS), based on a sample of hospital laboratories reporting to the European Antimicrobial Resistance network (EARS-Net), showed that in 2009 the percentage of carbapenem-resistant *K. pneumoniae* causing BSI was 1.3%; this percentage increased dramatically to 15.2% in 2010, to 26.7% in 2011 and to 34.3% in 2013, making *K. pneumoniae* the principal antibiotic-resistance threat in Italy [15-18]. Most other European Union (EU)/European Economic Area (EEA) countries do not have the endemic levels seen in Italy: the EU/EEA population-weighted mean of *K. pneumoniae* resistant to carbapenems was 4.6% in 2010 and 6.1% in 2016. In 2010, the percentage of carbapenem-resistant *K. pneumoniae* was very high only in Greece (59.5%) and Cyprus (17.9%), where it remains at high levels. Romania has also reported an endemic situation for CRE since 2012 [18].

To monitor and improve the prevention and control of these infections, in February 2013 the Italian Ministry of Health (MoH) instituted national surveillance for CPE through a circular letter (MoH DGPRE n°4968 26/02/2013), asking the 19 regional and the two provincial health authorities to report all cases of BSI due to carbapenem-resistant/-intermediate and/or carbapenemase-producing *K. pneumoniae* or *E. coli*. Other Enterobacteriaceae were not included in the surveillance, since these represent a small portion of all CPE in Italy [19].

In this paper, we report the results of this surveillance for the period from 2014 to 2017.

## Methods

### Case definition and diagnostic criteria for bloodstream infections due to carbapenemase-producing Enterobacteriaceae

All BSI due to *K. pneumoniae* or *E. coli* with at least one of the following characteristics: (i) non-susceptibility (i.e. resistant or intermediate) to the carbapenem antibiotics imipenem and/or meropenem obtained by a routine antibiotic susceptibility method; and (ii) production of carbapenemase identified by a phenotypic method [20] or by the detection of a carbapenemase gene by PCR or another molecular method.

In this case definition it was assumed that susceptibility to imipenem or meropenem is mainly due to the production of carbapenemases. In fact, for Enterobacteriaceae isolates obtained in Italy and, in particular, for *K. pneumoniae*, this is by far the most prevalent mechanism of carbapenem resistance [15,16,21].

### Information to be reported and data flow

Since April 2013, Local Health Units (LHUs) and hospitals have had to report all cases of BSI due to CPE (CPE-BSI), according to the previous definition, to the regional health authorities, to the MoH and to the Istituto Superiore di Sanità (ISS) within 7 days from the date of diagnosis, using a paper form [22]. This form has two sections: the first contains personal data used for infection control purpose and the second contains information about the notifier (the name and the city of the hospital or LHU and the municipality where hospital/LHU is located); patient demographics (sex, age at diagnosis, province of residence and nationality); patient location at onset of symptoms (hospital, other residential health structures, home, and for inpatients, hospital ward); presumptive source of BSI; patient outcome at the time of notification; and specimen data (date of sampling, pathogen detected, carbapenem-resistant status, phenotypic or genotypic confirmation of carbapenemase production and identification of the carbapenemase enzyme). Only section 2 is transferred to the central level, in order to maintain case anonymity.

Data collected at ISS are entered and stored in a dedicated Microsoft Access database.

### Statistical analysis

Data analysed and presented here were collected in the ISS database up to 18 July 2018 and refer to CPE-BSI occurring in the period from 1 January 2014 to 31 December 2017. Cases diagnosed earlier were excluded because of low adherence to the new surveillance system in 2013 [22]. We also excluded from analysis all BSI cases lacking date of diagnosis or indication of the microorganism involved or not meeting case definition (346/7,978, 4.3%). Data were summarised using frequency tables for categorical variables. Incidence rates (IR) were calculated at national level for the years 2014–17 either as number of notifications per 100,000 hospital patient days (HD) using data from the national hospital discharge registry (HDR) [23], or as number of notifications per 100,000 inhabitants using the Italian population as denominator [24-27]. The previous indicators were also calculated by specific age groups (5-year intervals) and by sex. We also calculated age-standardised [28] IR by calendar year. When calculating specific and adjusted IR, cases with unspecified sex (1.1%) or age (3.0%) were randomly imputed based on the relative sex and age distribution of the other cases.

Data were analysed using Stata, version 13.0 (Stata Corporation, College Station, TX, US).

## Results

In the period 2014–17, 330 LHUs or hospitals reported 7,632 CPE-BSI from all Italian regions and autonomous provinces. In particular, LHUs or hospitals reporting at least one case every year were 153, 196, 211 and 214 in 2014, 2015, 2016, and 2017, respectively. Most of the cases (7,490, 98.1%) were due to *K. pneumoniae,* while *E. coli* was reported in only 142 (1.9%) cases. Patients were mainly male (62.7%) and more than 70% were ≥ 60 years old. Less than 4% of the cases had non-Italian nationality (Table 1) and of these, more than 40% were from eastern Europe. Specifically, the largest group of patients with non-Italian nationality were from Romania (54/271, 19.9%), Albania (23/271, 8.5%), Morocco (17/271, 6.3%) and Ukraine (11/271, 4.1%); the remaining cases came from 46 other countries.

**Table 1:** Main characteristics of cases of bloodstream infection due to carbapenemase-producing Enterobacteriaceae reported to the national surveillance system, Italy, 2014–2017 (n = 7,632)

Characteristics		N	%
Pathogen	*Klebsiella pneumoniae*	7,490	98.1
	*Escherichia coli*	142	1.9
Sex^a^	Female	2,817	37.3
	Male	4,731	62.7
Age group (years)^a^	0–19	101	1.4
	20–39	411	5.6
	40–59	1,642	22.2
	60–79	3,677	49.7
	≥ 80	1,569	21.2
Nationality	Italian	7,631	96.4
	Other	271	3.6
Patient location at symptom onset^a^	Hospital	6,386	87.2
	Other^b^	937	12.8
Total		7,632	100

IR: incidence rate.

^a ^For 84 (1.1%) cases, sex was not indicated; for 232 (3.0%) cases, age was not reported; for 309 (4.0%) cases, patient location was not indicated.

^b ^Home or long-term care facility.

For the majority of patients for whom information was available, the onset of symptoms occurred in a hospital setting (87.2%); symptoms occurred at home (9.9%) or in a long-term care facility (2.9%) for a minority of patients. When BSI onset occurred in hospital, patients were more commonly in intensive care units (ICU) (2,430/6,401, 38.0%), in general medicine wards (881/6,401, 13.8%) or in surgical wards (725/6,401, 11.3%). Other wards were reported less frequently, and accounted for 36.9% (2,365/6,401) of cases (data not shown).

**Figure 1:**
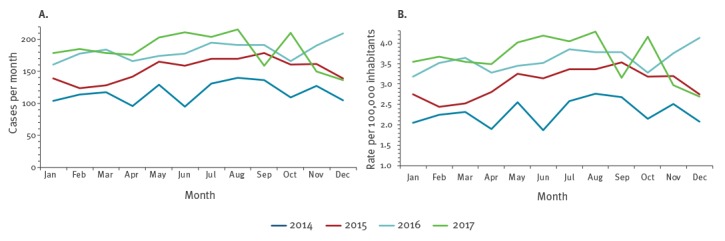
(A) Frequency and (B) incidence rate per 100,000 inhabitants by month and year of bloodstream infections due to carbapenemase-producing Enterobacteriaceae reported to the national surveillance system, Italy, 2014–2017

Figure 1 shows the frequency and crude IR (per 100,000 inhabitants) of CPE-BSI cases distributed by month and year of diagnosis. We observed an increase in the annual number of reported cases, as well as in the IR over the period analysed. The annual number, the crude IR and the age-standardised IR in 2014 were 1,403, 2.31 and 2.10, respectively; in 2017 these numbers raised to 2,208, 3.64 and 3.30, respectively. Overall, the IR increased significantly during the study period (incidence rate ratio (IRR) = 1.16, 95% confidence interval (CI): 1.13–1.18); however, the IRs in 2016 and 2017 were very similar (IRR = 1.01, 95% CI: 0.95–1.07, p = 0.67). Similar results were obtained adjusting for age and sex.

The CPE-BSI IR among hospitalised patients was 2.97 per 100,000 HD, with an increasing trend from 2.12 in 2014 to 3.45 in 2017 (data not shown).

**Figure 2:**
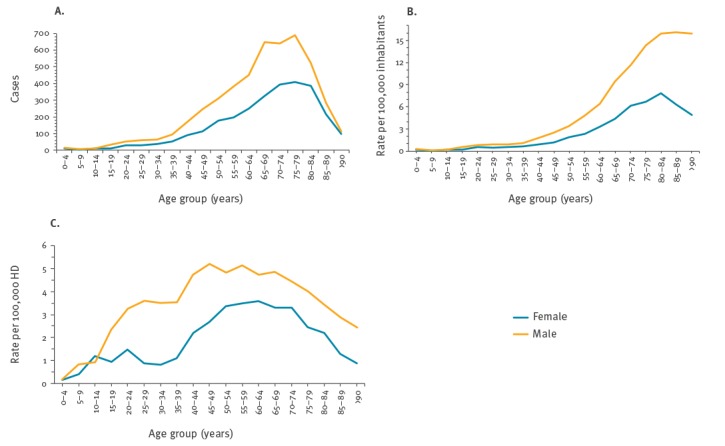
(A) Frequency, (B) incidence rate per 100,000 inhabitants and (C) incidence rate per 100,000 hospital patient days of bloodstream infection cases due to carbapenemase-producing Enterobacteriaceae stratified by age group and sex reported to the national surveillance system, Italy, 2014–2017

Figure 2 shows the frequency of CPE-BSI cases (panel A), the IR per 100,000 inhabitants (panel B) and the IR per 100,000 HD (panel C) stratified by age group and sex reported in the period 2014–17. We observed that the number of cases slightly increased with age until 40 years, then cases dramatically increased with a peak at 65–79 years; cases sharply declined in the oldest age groups. The number of cases was always higher in males than in females for all age groups (Figure 2, panel A).

When considering the IRs per 100,000 inhabitants by age group and sex (Figure 2, panel B), we observed similar IRs in males and females up to the 35–39 years age group, higher IRs in males than in females in the older age groups and then a decreasing trend in females after the 80–84 years age group.

When considering the specific IR per 100,000 HD (IR-HD) by age group and sex (Figure 2, panel C) among hospitalised patients, we observed that the IR-HD was higher among men in each age group compared with females with the exception of the 10–14 years age group. Overall, the IR-HD did not show a continuous increase with age. The highest IR-HD was observed for the 50–54 and 55–59 years age groups, with a decreasing trend starting from the 70–74 years age group.

Mostly, the reported source of the CPE-BSI was a central or peripheral venous catheter (CVC/PVC) (1,376/5,754, 23.9%;) or a urinary tract infection (UTI) (1,216/5,754, 21.1%;). A primary infection was reported in 20.4% (1,174/5,754) of cases; less frequently, the source of the CPE-BSI was associated with pneumonia (including ventilator-associated pneumonia) (926/5,754, 16.1%), abdominal infections (731/5,754, 12.7%), non-surgical site infections (NSSI) (172/5,754, 3.0%), or surgical site infections (SSI) (159/5,754, 2.8%) (data not shown).

When stratifying data on source of CPE-BSI by sex, we observed significant differences between males and females: origin from NSSI or SSI was significantly more frequently reported in females than in males (respectively 3.7% 77/2,101 vs 2.6% 95/3,606, p = 0.03 and 3.7% 77/2,101 vs 2.3% 82/3,606, p < 0.01). On the other hand, origin from UTI or pneumonia was significantly more frequently reported in males than in females (respectively 22.5% 812/3,606 vs 18.7% 393/2,101, p < 0.01 and 17.2% 620/3,606 vs 14.1% 296/2,101, p < 0.01).

At the time of reporting, a high percentage of cases was still in hospital (5,247/6,869, 76.4%), 6.7% (457/6,869) had been discharged, and the remaining 17.0% (1,165/6,869) was reported to have died. Mortality was significantly associated with age (p < 0.01); in particular, mortality was higher in children 0–9 years old (5/31, 16.1%) and in the elderly aged ≥ 75 years (528/2,428, 21.7%). Using univariate analysis, women showed a higher mortality compared with men (18.1% vs 16.4%, 460/2,538 vs 700/4,269 respectively), although this was not statistically significant (p = 0.07). When performing a multiple logistic model adjusting simultaneously for age and sex, age was still associated with death significantly (p < 0.01) while there was not a significant association with sex (data not shown).

A carbapenemase enzyme identified in the CPE strains isolated from BSI was reported in 60.4% (4,612/7,632) of cases only. There was no association with the lack of carbapenemase enzyme identification with age and sex of the patient and with the year of diagnosis. Carbapenemase enzymes were detected mostly by phenotypic tests only (3,004/4,612, 65.1%); genotypic tests only or both tests were applied in a minority of cases (625/4,612 and 983/4,612, 13.6% and 21.3%, respectively). However, over the years there was a significant increase of the detection of carbapenemase enzymes using genotypic tests (from 24.7%, 218/881, in 2014 to 45.1%, 596/1,321 in 2017). In most cases the enzyme reported was *K. pneumoniae* carbapenemase (KPC) (in 95.2% of *K. pneumoniae* and 81.4% of *E. coli)*. Metallo-beta-lactamases (MBL) were reported in 2.1% of cases, and carbapenem-hydrolysing oxacillinase-48 (OXA-48) in 1.2%. Associations between MBL and KPC (0.9%) or MBL and OXA-48 (0.3%) were also rarely identified. When genotypic tests were applied to identify the MBL genes, those detected were mainly Verona integron-encoded metallo-beta-lactamase (VIM) (65/86, 75.6%) followed by New Delhi metallo-beta-lactamase (NDM) (21/86, 24.4%)(Table 2). NDM was reported only in the years 2016–17, mainly in *K. pneumoniae* (20/21, 95.2%), and often in association with OXA-48.

**Table 2:** Type of carbapenemase enzyme detected in carbapenemase-producing Enterobacteriaceae isolates from bloodstream infections reported to the national surveillance system, Italy, 2014–2017

Carbapenemase enzyme	*Klebsiella pneumoniae*		*Escherichia coli*		Total	
	N	%	N	%	N	%
KPC	4,323	95.2	57	81.4	4,380	95.0
MBL^a^	87	1.9	12	17.1	99	2.1
KPC + MBL^b^	43	0.9	0	0.0	43	0.9
OXA-48	55	1.2	1	1.4	56	1.2
MBL^c^ + OXA-48	15	0.3	0	0.0	15	0.3
KPC + OXA-48	3	0.1	0	0.0	3	0.1
ND^d^	16	0.4	0	0.0	16	0.3
Not indicated	2,948	–	72	–	3,020	–
Total	7,490	–	142	–	7,632	–

KPC: *Klebsiella pneumoniae* carbapenemase; MBL: metallo-beta-lactamase; ND: not determined; NDM: New Delhi metallo-beta-lactamase; OXA-48: carbapenem-hydrolysing oxacillinase-48; VIM: Verona integron-encoded metallo-beta-lactamase.

^a^ Genotyping available for 59 MBLs: 51 VIM (47 in *K. pneumoniae*; 4 in *E. coli*) and 8 NDM (7 in *K. pneumoniae*; 1 in *E. coli*).

^b^ Genotyping available for 13 MBLs: 11 VIM and 2 NDM (all in *K. pneumoniae*).

^c^ Genotyping available for 14 MBLs: 3 VIM and 11 NDM (all in *K. pneumoniae*).

^d^ Not determined (discrepancy between genotypic and phenotypic results).

Percentages were calculated excluding those isolates without enzyme identification.

## Discussion

During the period under analysis, the CPE-BSI crude IR initially increased until 2016 then reached a plateau, suggesting a stable but highly endemic situation. These results confirm the strong public health burden of CPE infections in Italy, as already indicated by the EuSCAPE project, based on sentinel hospitals [15,17], or by data from single hospitals/regions [21,29,30].

The results of the countrywide surveillance showed a high incidence of CPE-BSI in the Italian population, especially in men aged 65 years and older. The microbiological results of the surveillance showed predominance of KPC-producing *K. pneumoniae* and marginal presence of other carbapenemase enzymes such as MBL, confirming previous findings [8,31].

CPE-BSI cases more often occurred in hospitalised patients admitted to ICU or to general medicine wards, mainly associated with the presence of invasive devices (e.g. CVC/PVC).

The reason for the higher incidence of CPE-BSI in males than in females is not clear, but it is in accordance with the observation that the incidence of BSI/sepsis by all pathogens is higher in males than in females [32]. It is of note that in several studies this difference was found specifically for BSI/sepsis due to *Klebsiella species* [33-35]. Confirmation of this finding can also be obtained examining the European Centre for Disease Prevention and Control Atlas database, where the higher proportion of carbapenem-resistant *K. pneumoniae* in males is particularly evident in countries at high prevalence of carbapenem resistance such as Romania and Italy. Although there is no clear hypothesis to explain this difference, the higher frequency of sepsis in males has been associated with a genetic predisposition [36]. It is acknowledged that marked differences exist between males and females in the incidence of various diseases, including infections. Such differences may find an explanation in immunological responses, in physiological and anatomical differences influencing exposure and transmission of microorganisms, or even in behavioural factors influencing exposure to microorganisms or access to healthcare [37].

In our study, we observed an increase of CPE-BSI IR as age increased. This increase, particularly in patients aged 65 years and older, could be related to the higher vulnerability of the elderly [38], which could determine higher frequency and longer hospital stay compared with younger patients. The association with age had already been described in another study published in 2007 on all type of bacteraemia [33].

Considering IR for hospitalised patients only, and based on HD, the association with age was less strong. In fact, while we confirmed higher IR-HD in males than in females, the IR-HD did not show a continuous increase with age, being higher in patients aged 50–59 years and decreasing in those aged 70 years and over. The decreasing trend of CPE-BSI in hospitalised elderly patients could be related to the admission of these patients to wards with less intensive levels of care (e.g. long-term care/geriatrics), with a possible lower risk of acquiring CPE infections. Unfortunately, data from HDR provided by the MoH did not contain details about all the ward units to which patients were admitted throughout their hospital stay.

The greater number of deaths observed in the extreme ages of life could be due to the limited ability of newborns to mount an efficient immune response [39] and to the poor response of the immune system [38] and the presence of comorbidities in elderly patients.

Because most CPE-BSI cases occurred in Italian patients, the impact of foreign or migrant populations seems to be marginal. However, we were not able to calculate IR stratified by nationality to better disentangle the data.

This study has some limitations. We cannot exclude the possibility that the increase in CPE-BSI IR over the 4-year period could be due at least in part to the progressive adherence of the LHUs and the hospitals to the surveillance system. Under-reporting is likely present, as suggested by differences in the number of cases when comparing data from this surveillance system with those from other sources such as antibiotic resistance surveillance AR-ISS, regional reports or scientific publications [22], (Supplement S1). Another limitation of this study is that this national surveillance system is based on a broad case definition not requiring the detection of carbapenemase production. In fact, ca 40% of the CPE-BSI cases reported do not contain information about the phenotypic or genotypic detection of the resistance mechanism involved, so the system cannot recognise isolates with resistance mechanisms other than carbapenemase production (e.g. reduction of membrane permeability in combination with overexpression of AmpC enzymes or ESBLs) that are included in the CPE surveillance. However, the predominance of carbapenemase producing *K. pneumoniae* among carbapenem non-susceptible isolates reported by previous studies in Italy [8,16] suggests that the isolates in which carbapenemase production was not specified were mainly CPE. Lastly, the mortality associated with CPE infection may have been underestimated because there was no planned follow up of the patients.

The surveillance results show that CPE-BSI represent a high burden of serious antibiotic-resistant infections and should be considered a threat to patient safety in Italy and also in other countries due to the cross-border mobility of patients [40]. The situation suggests there is a need for urgent interventions by the Italian health authorities.

To control the spread of CPE infections, the circular letter that instituted the CPE surveillance (MoH DGPRE n°4968 26/02/2013) also contained specific recommendations such as application of infection control protocols including screening of patients and isolation of cases, especially in intensive care and other high-risk wards. However, the burden of antibiotic-resistant infections in Italy remains a serious issue and additional efforts in infection control should be made. To this end, in November 2017 the Italian MoH, in accordance with the regional authorities, issued the first strategic Italian plan to fight antimicrobial resistance with a one-health approach [41]. Several activities are planned over a 3-year period to prevent and control antibiotic-resistant infections including a reinforcement of surveillance, prevention and control of healthcare-associated infections, antimicrobial stewardship, training, communication campaigns and research.

Within this plan, BSI-CPE surveillance plays a central role in monitoring the impact of the implementation of the various activities included in the national plan at regional and hospital level.

## Supplementary Data

421000 Supplement S1
