# Supplementary material for: Bloodstream infections due to carbapenemase-producing Enterobacteriaceae in Italy: results from nationwide surveillance, 2014 to 2017
Source: Euro Surveill. 2019 Jan 31;24(5):1800159. doi: 10.2807/1560-7917.ES.2019.24.5.1800159 (PMC6386214; doi:10.2807/1560-7917.ES.2019.24.5.1800159)
Supplement: Supplement S1 [file 1800159_IACCHINI_Supplement.pdf]

This supplementary material is hosted by *Eurosurveillance* as supporting information alongside the article 'Bloodstream infections due to carbapenemase-producing Enterobacteriaceae in Italy: results from nationwide surveillance, 2014 to 2017' on behalf of the authors who remain responsible for the accuracy and appropriateness of the content. The same standards for ethics, copyright, attributions and permissions as for the article apply. *Eurosurveillance* is not responsible for the maintenance of any links or email addresses provided therein.

Supplement. Number of carbapenem-resistant *K. pneumoniae* (panel A) and *E. coli* (panel B) isolates, Italy, 2014-2016. Comparison between data from the national surveillance system for carbapenemase-producing Enterobacteriaceae (CPE) and data from other sources.

A

**region**	**carbapenem-resistant *K. pneumoniae* isolates**						
	**2014**		**2015**		**2016**		
	**national surveillance**	***other data source***	**national surveillance**	***other data source***	**national surveillance**	***other data source***	**type of other data source**
Piemonte	248	*234*	239	*244*	220	*283*	regional surv.[c]
Emilia-Romagna	147	*129*	222	*260*	265	*255*	regional report [d]
Campania	61	*162* [a]	61	*198* [a]	106	*305* [a]	regional report [e]
Toscana	111	*266* [b]	90	*261* [b]	112	*298* [b]	regional report [f]

[a] the regional antimicrobial resistance report shows this number of *K. pneumoniae* or *E. coli* isolates from blood and liquor as resistant to meropenem and/or imipenem; here we report the highest number of cases resistant to either antibiotic.

[b] the regional antimicrobial resistance report shows this number of *K. pneumoniae* or *E. coli* isolates from blood as resistant to meropenem and/or imipenem; here we report the highest number of cases resistant to either antibiotic.

[c] https://www.seremi.it/viz-condizioni/Antibioticoresistenze

[d] http://assr.regione.emilia-romagna.it/it/servizi/pubblicazioni/rapporti-documenti/sorveglianza-antibioticoresistenza-uso-antibiotici-rapporto-2016

[e] http://www.regione.campania.it/regione/it/tematiche/antibiotico-resistenza-ed-infezioni-correlate-all-assistenza-64in/antibiotico-resistenza-ed-infezioni-correlate-all-assistenza-r11p

[f] https://www.ars.toscana.it/it/pubblicazioni/rapporti-relazioni-e-sintesi/2017/3855-report-2016-su-antibiotico-resistenza-e-uso-di-antibiotici-in-toscana.html

B

**region**	**carbapenem-resistant *E. coli* isolates**						
	**2014**		**2015**		**2016**		
	**national surveillance**	***other data source***	**national surveillance**	***other data source***	**national surveillance**	***other data source***	**type of other data source**
Piemonte	4	*4*	6	*7*	4	*4*	regional surv.[c]
Emilia-Romagna	1	*4*	5	*6*	8	*8*	regional report [d]
Campania	0	*3* [a]	0	*5* [a]	1	*9* [a]	regional report [e]
Toscana	1	*2* [b]	2	*5* [b]	2	*4* [b]	regional report [f]

[a] the regional antimicrobial resistance report shows this number of *K. pneumoniae* or *E. coli* isolates from blood and liquor as resistant to meropenem and/or imipenem; here we report the highest number of cases resistant to either antibiotic.

[b] the regional antimicrobial resistance report shows this number of *K. pneumoniae* or *E. coli* isolates from blood as resistant to meropenem and/or imipenem; here we report the highest number of cases resistant to either antibiotic.

[c] https://www.seremi.it/viz-condizioni/Antibioticoresistenze

[d] http://assr.regione.emilia-romagna.it/it/servizi/pubblicazioni/rapporti-documenti/sorveglianza-antibioticoresistenza-uso-antibiotici-rapporto-2016

[e] http://www.regione.campania.it/regione/it/tematiche/antibiotico-resistenza-ed-infezioni-correlate-all-assistenza-64in/antibiotico-resistenza-ed-infezioni-correlate-all-assistenza-r11p

[f] https://www.ars.toscana.it/it/pubblicazioni/rapporti-relazioni-e-sintesi/2017/3855-report-2016-su-antibiotico-resistenza-e-uso-di-antibiotici-in-toscana.html
